# Supplementary figures and images for: Genome-wide association analysis identifies genetic correlates of immune infiltrates in solid tumors
Source: PLoS One. 2017 Jul 27;12(7):e0179726. doi: 10.1371/journal.pone.0179726 (PMC5531551; doi:10.1371/journal.pone.0179726)

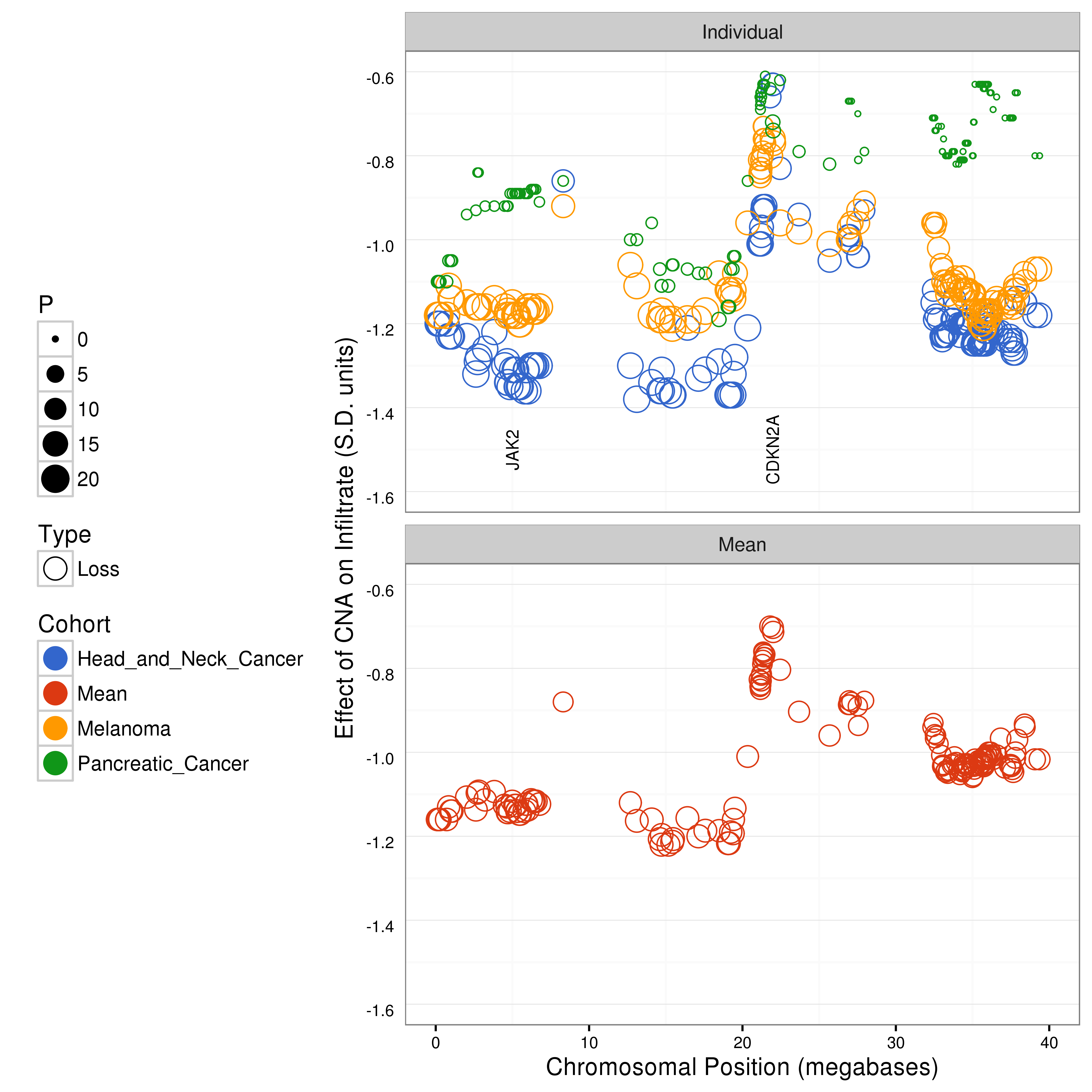

Supplement: S1 Fig — Chromosomal location is displayed on the horizontal axis, and the magnitude of effect of copy number change on CD8+ T cell estimates (rather than -logP, as in the main figures) is displayed on the vertical axis. Each data point represents the result for a given locus, with significance (negative log(10) of P value) indicated by size of the data point. The unit of effect size is the change in TCD8 signature score (units of standard deviation of signature score across all TCGA tumors) per (log2) unit of GISTIC copy number change. Individual panel: association between loss of a given chromosomal region and CD8+ T cell estimates in melanoma, pancreatic, head-neck cancer. Mean panel: a combined analysis across the three cohorts (mean effect size). (TIFF) [file pone.0179726.s001.tiff]
